# Supplementary material for: The motor domain of the kinesin Kip2 promotes microtubule polymerization at microtubule tips
Source: J Cell Biol. 2023 Apr 24;222(7):e202110126. doi: 10.1083/jcb.202110126 (PMC10130750; doi:10.1083/jcb.202110126)
Supplement: Table S1 — shows quantification of preanaphase astral microtubule length and dynamics in living cells. [file JCB_202110126_TableS1.docx]

**Table S1.** Quantification of preanaphase astral microtubule length and dynamics in living cells.

| ***Plus-end marker*** | **Bik1-3xGFP** | | |
| --- | --- | --- | --- |
| ***Strains*** | WT | *kip2Δ* | *kip2-ΔT* |
| ***aMT length*** *(µm)* | 2.1 ± 0.1  (n = 509) | 0.2 ± 0.1^****^  (n = 2001) | 0.4 ± 0.1^****^  (n = 918) |
| ***Growth speed***  *(µm min^-1^)* | 2.0 ± 1.8 | 1.5 ± 0.9* | 2.0 ± 2.1 (n.s.) |
| ***Shrinkage speed*** *(µm min^-1^)* | 3.1 ± 1.5 | 4.2 ± 2.1** | 3.8 ± 2.2* |
| ***Cat. frequency*** *(events min^-1^)* | 2.8  (n = 531) | 5.1  (n = 306) | 3.9  (n = 279) |
| ***Res. frequency***  *(events min^-1^)* | 1.0  (n = 216) | 1.7  (n = 73) | 1.7  (n = 108) |

Average maximal 3D microtubule lengths over the time-lapse series were measured. All lengths of microtubules below the detection limit (666.7 nm owing to the microscope resolution) were marked as 0 µm. See Materials and methods for details. n.d. refers to not determined. For microtubule length, statistical significances of difference from respective WT control were tested with one-way ANOVA and shown in the table. **** p < 0.0001, *** p < 0.001, ** p < 0.01, * p < 0.05, n.s., not significant. All data are reported as mean ± SD and are available in **Data S1**.
