# Supplementary material for: The motor domain of the kinesin Kip2 promotes microtubule polymerization at microtubule tips
Source: J Cell Biol. 2023 Apr 24;222(7):e202110126. doi: 10.1083/jcb.202110126 (PMC10130750; doi:10.1083/jcb.202110126)
Supplement: Table S2 — shows quantification of microtubule length and dynamics in vitro with 7 µM porcine tubulin. [file JCB_202110126_TableS2.docx]

**Table S2.** Quantification of microtubule length and dynamics in vitro with 7 µM porcine tubulin.

|  | **Control** | **Kip2-WT** | | | **Kip2-P1^-^** | | |
| --- | --- | --- | --- | --- | --- | --- | --- |
| **[Kip2] nM** | 0 | 2 | 4 | 8 | 2 | 4 | 8 |
| ***Length***  *(µm)* | 1.2 ± 0.7  (n = 15) | 5.8 ± 1.8  (n = 107) | 10.5 ± 2.2  (n = 117) | 13.6 ± 2.3  (n = 95) | 0.8 ± 0.2 (n = 21) | 3.2 ± 1.9  (n = 104) | 3.4 ± 2.2  (n = 86) |
| ***Growth speed***  *(µm min^-1^)* | 0.6 ± 0.4  (n = 19) | 8.3 ± 0.9  (n = 107) | 9.4 ± 0.3  (n = 121) | 10.0 ± 1.8  (n = 95) | 0.7 ± 2.0  (n = 136) | 3.9 ± 3.3  (n = 141) | 4.6 ± 3.8  (n = 112) |
| ***Shrinkage speed*** *(µm min^-1^)* | 20.0 ± 8.4  (n = 3) | 13.3 ± 6.2  (n = 17) | 9.8 ± 5.8  (n = 6) | - | - | 12.5 ± 8.3  (n = 65) | 12.4 ± 6.4  (n = 40) |
| ***Cat. frequency*** *(events min^-1^)* | 0.5 ± 0.3  (n = 13) | 0.03 ± 0.07  (n = 107) | 0.005 ± 0.02  (n = 121) | - | 0.3 ± 0.2  (n = 21) | 0.2 ± 0.2  (n = 21) | 0.2 ± 0.2  (n = 80) |
| ***Res. frequency***  *(events min^-1^)* | 0 ± 0  (n = 2) | 3.2 ± 4.6  (n = 13) | 4.4 ± 2.0  (n = 121) | - | - | 0.82 ± 2.4  (n = 58) | 1.2 ± 2.1  (n = 18) |

All data are reported as mean ± SD and are available in **Data S1**.
