# Supplementary material for: The motor domain of the kinesin Kip2 promotes microtubule polymerization at microtubule tips
Source: J Cell Biol. 2023 Apr 24;222(7):e202110126. doi: 10.1083/jcb.202110126 (PMC10130750; doi:10.1083/jcb.202110126)
Supplement: Table S3 — lists all recombinant proteins used in in vitro studies. [file JCB_202110126_TableS3.docx]

**Table S3.**

All recombinant proteins used in in vitro studies.

| **Name used** | **Composition** | **Used in** |
| --- | --- | --- |
| Kip2-NMD | Kip2[504-706] | Figure 2BCDE |
| Kip2-NMD-∆T | Kip2[504-645] | Figure 2E |
| Bik1-CC | Bik1[182-396] | Figure 2DEF |
| Kip2-MD | Kip2[100-503] | Figure 5A |
| MBP-Kip2-MD-mCherry | MBP-Kip2[71-560]-mCherry | Figure 5C |
| MBP-Kip2-MD-P1^-^-mCherry | MBP-Kip2[71-560]-K294AR296A-mCherry | Figure 5D |
| MBP-GFP | MBP-GFP | Figure 6A-E |
| Kip2-WT | MBP-Kip2[1-560]-RFP | Figure 6A-E, Figure S5CD |
| Kip2-P1^-^ | MBP-Kip2[1-560]-K294AR296A-RFP | Figure 6A-E, Figure S5CD |
