# Supplementary material for: The motor domain of the kinesin Kip2 promotes microtubule polymerization at microtubule tips
Source: J Cell Biol. 2023 Apr 24;222(7):e202110126. doi: 10.1083/jcb.202110126 (PMC10130750; doi:10.1083/jcb.202110126)
Supplement: Table S4 — shows yeast strains used in this study. [file JCB_202110126_TableS4.docx]

Table S4.

Yeast strains used in this study.

**­**

| **Yeast (yYB)**  **Strain number** | **Mating**  **type** | **Genotype** | **Background** | **Source** |
| --- | --- | --- | --- | --- |
| 11068 | alpha | *Bik1-3xGFP:hyg Spc72-GFP:His3MX*  *ura3-52 his3Δ200 leu2 lys2-801 trp1Δ63 Ade2+* | S288C | (Stangier et al., 2018) |
| 11069 | a | *Bik1-3xGFP:hyg Spc72-GFP:His3MX*  *ura3-52 his3Δ200 leu2 lys2-801 trp1Δ63 Ade2+* | S288C | (Stangier et al., 2018) |
| 12781 | alpha | *Bik1-3xGFP:hyg Spc72-GFP:His3MX*  *kip2∆::Kip2-∆T(1-644):TRP*  *ura3-52 his3Δ200 leu2 lys2-801 trp1Δ63 Ade2+* | S288C | this study |
| 11763 | alpha | *Bik1-3xGFP:hyg Spc72-GFP:His3MX kip2::hphNT1*  *ura3-52 his3Δ200 leu2 lys2-801 trp1Δ63 Ade2+* | S288C | this study |
| 15100 | a | *Kip2-3xsfGFP:KanMX Spc42-mCherry:NatMX*  *ura3-52 his3Δ200 leu2 lys2-801 trp1Δ63 Ade2+* | S288C | (Chen et al., 2019b) |
| 15109 | a | *Kip2-3xsfGFP:KanMX Spc42-mCherry:NatMX bik1::hphNT1*  *ura3-52 his3Δ200 leu2 lys2-801 trp1Δ63 Ade2+* | S288C | this study |
| 15101 | alpha | *kip2∆::Kip2-∆T-3xsfGFP:KanMX Spc42-mCherry:NatMX*  *ura3-52 his3Δ200 leu2 lys2-801 trp1Δ63 Ade2+* | S288C | this study |
| 11946 | a | *kip2∆::Kip2-NMD(510-706)-mNeonGreen:NatMX*  *Spc72-GFP:His3MX*  *ura3-52 his3Δ200 leu2 lys2-801 trp1Δ63 Ade2+* | S288C | this study |
| 15472 | a | *kip2∆::Kip2-NMD(510-706)-3xsfGFP:KanMX*  *Spc42-mCherry:NatMX*  *ura3-52 his3Δ200 leu2 lys2-801 trp1Δ63 Ade2+* | S288C | this study |
| 14713 | a | *kip2∆::Kip2-NMD-∆T(510-644)-3xsfGFP:KanMX*  *Spc42-mCherry:NatMX kip3::URA*  *ura3-52 his3Δ200 leu2 lys2-801 trp1Δ63 Ade2+* | S288C | this study |
| 14663 | a | *kip2∆::Kip2-NMD(510-706)-3xsfGFP:KanMX*  *Spc42-mCherry:NatMX kip3::URA*  *ura3-52 his3Δ200 leu2 lys2-801 trp1Δ63 Ade2+* | S288C | this study |
| 15473 | a | *kip2∆::Kip2-NMD(510-706)-3xsfGFP:KanMX*  *Spc42-mCherry:NatMX bik1::NatMX*  *ura3-52 his3Δ200 leu2 lys2-801 trp1Δ63 Ade2+* | S288C | this study |
| 9806 | a | *Kip2-3xsfGFP:KanMX4 Spc72-GFP:His3MX*  *ura3-52 his3Δ200 leu2 lys2-801 trp1Δ63 Ade2+* | S288C | (Chen et al., 2019b) |
| 11046 | a | *Kip2-3xsfGFP:KanMX4 Spc72-GFP:His3MX bik1::hphNT1*  *ura3-52 his3Δ200 leu2 lys2-801 trp1Δ63 Ade2+* | S288C | this study |
| 10795 | a | *kip2∆::Kip2-∆T-3xsfGFP:KanMX Spc72-GFP:His3MX*  *ura3-52 his3Δ200 leu2 lys2-801 trp1Δ63 Ade2+* | S288C | this study |
| 14269 | a | *kip2∆::Kip2-6HA:NatMX*  *ura3-52 his3Δ200 leu2 lys2-801 trp1Δ63 Ade2+* | S288C | (Chen et al., 2019b) |
| 14267 | a | *kip2∆::Kip2-∆T-6HA:NatMX*  *ura3-52 his3Δ200 leu2 lys2-801 trp1Δ63 Ade2+* | S288C | this study |
| 14271 | a | *kip2∆::Kip2-6HA:NatMX bik1::hphNT1*  *Kip3-3xsfGFP:KanMX*  *ura3-52 his3Δ200 leu2 lys2-801 trp1Δ63 Ade2+* | S288C | this study |
| 11553 | a | *Kip2-mNeonGreen:NatMX Spc72-GFP:His3MX*  *ura3-52 his3Δ200 leu2 lys2-801 trp1Δ63 Ade2+* | S288C | this study |
| 11642 | a | *Kip2-mNeonGreen:NatMX Spc72-GFP:His3MX bik1::hphNT1*  *ura3-52 his3Δ200 leu2 lys2-801 trp1Δ63 Ade2+* | S288C | this study |
| 15105 | a | *Kip2-G374A-3xsfGFP:KanMX Spc42-mCherry:NatMX*  *ura3-52 his3Δ200 leu2 lys2-801 trp1Δ63 Ade2+* | S288C | (Chen et al., 2019b) |
| 15104 | a | *Kip2-G374A-3xsfGFP:KanMX Spc42-mCherry:NatMX bik1::NatMX*  *ura3-52 his3Δ200 leu2 lys2-801 trp1Δ63 Ade2+* | S288C | this study |
| 15334 | a | *Kip2-K294AR296A-3xsfGFP:KanMX Spc42-mCherry:NatMX*  *ura3-52 his3Δ200 leu2 lys2-801 trp1Δ63 Ade2+* | S288C | this study |
| 15333 | a | *Kip2-R384AR385A-3xsfGFP:KanMX Spc42-mCherry:NatMX*  *ura3-52 his3Δ200 leu2 lys2-801 trp1Δ63 Ade2+* | S288C | this study |
| 15332 | alpha | *Kip2-R446A-3xsfGFP:KanMX Spc42-mCherry:NatMX*  *ura3-52 his3Δ200 leu2 lys2-801 trp1Δ63 Ade2+* | S288C | this study |
| 12566 | alpha | *Bik1-3xGFP:hyg Spc72-GFP:His3MX*  *kip2∆::Kip2-∆N(91-706):TRP*  *ura3-52 his3Δ200 leu2 lys2-801 trp1Δ63 Ade2+* | S288C | this study |
| 12395 | alpha | *Spc72-GFP:His3MX kip2::hphNT1*  *kip3∆::Kip3(1-480)-Kip2(510-706)-mNeonGreen:NatMX*  *ura3-52 his3Δ200 leu2 lys2-801 trp1Δ63 Ade2+* | S288C | this study |
| 13234 | diploid | *Bik1-3xGFP:hyg / Bik1-3xGFP:hyg*  *Spc72-GFP:His3MX / Spc72-GFP:His3MX*  *ura3-52 his3Δ200 leu2 lys2-801 trp1Δ63 Ade2+* | S288C | this study |
| 13251 | diploid | *Bik1-3xGFP:hyg / Bik1-3xGFP:hyg*  *Spc72-GFP:His3MX / Spc72-GFP:His3MX*  *kip2::hphNT1 / KIP2*  *ura3-52 his3Δ200 leu2 lys2-801 trp1Δ63 Ade2+* | S288C | this study |
| 15099 | diploid | *Bik1-3xGFP:hyg / Bik1-3xGFP:hyg*  *Spc72-GFP:His3MX / Spc72-GFP:His3MX*  *KIP2 / kip2∆::Kip3(1-480)-Kip2(510-706)-6HA:NatMX*  *ura3-52 his3Δ200 leu2 lys2-801 trp1Δ63 Ade2+* | S288C | this study |
| 15363 | diploid | *Kip2-3xsfGFP:KanMX / Kip2-mCherry:hphNT1*  *Spc42-mCherry:NatMX / SPC42*  *ura3-52 his3Δ200 leu2 lys2-801 trp1Δ63 Ade2+* | S288C | this study |
| 15359 | diploid | *Kip2-R384AR385A-3xsfGFP:KanMX / Kip2-mCherry:hphNT1*  *Spc42-mCherry:NatMX / SPC42*  *ura3-52 his3Δ200 leu2 lys2-801 trp1Δ63 Ade2+* | S288C | this study |
| 15360 | diploid | *Kip2-K294AR296A-3xsfGFP:KanMX / Kip2-mCherry:hphNT1*  *Spc42-mCherry:NatMX / SPC42*  *ura3-52 his3Δ200 leu2 lys2-801 trp1Δ63 Ade2+* | S288C | this study |
| 15361 | diploid | *Kip2-R446A-3xsfGFP:KanMX / Kip2-mCherry:hphNT1*  *Spc42-mCherry:NatMX / SPC42*  *ura3-52 his3Δ200 leu2 lys2-801 trp1Δ63 Ade2+* | S288C | this study |
| 15384 | alpha | *Kip2-K294AR296A-3xsfGFP:KanMX Spc72-GFP:His3MX*  *kip3::URA*  *ura3-52 his3Δ200 leu2 lys2-801 trp1Δ63 Ade2+* | S288C | this study |
| 11711 | a | *Kip2-3xsfGFP:KanMX Spc72-GFP:His3MX kip3::URA*  *ura3-52 his3Δ200 leu2 lys2-801 trp1Δ63 Ade2+* | S288C | this study |
| 12564 | alpha | *Bik1-3xGFP:hyg Spc72-GFP:His3MX*  *kip2∆::Kip2-wt:TRP*  *ura3-52 his3Δ200 leu2 lys2-801 trp1Δ63 Ade2+* | S288C | (Chen et al., 2019b) |
| 14991 | alpha | *Bik1-3xGFP:hyg Spc72-GFP:His3MX*  *kip2∆::Kip2-K294AR296A:TRP*  *ura3-52 his3Δ200 leu2 lys2-801 trp1Δ63 Ade2+* | S288C | this study |
| 14989 | alpha | *Bik1-3xGFP:hyg Spc72-GFP:His3MX*  *kip2∆::Kip2-R384AR385A:TRP*  *ura3-52 his3Δ200 leu2 lys2-801 trp1Δ63 Ade2+* | S288C | this study |
| 14994 | alpha | *Bik1-3xGFP:hyg Spc72-GFP:His3MX*  *kip2∆::Kip2-R446A:TRP*  *ura3-52 his3Δ200 leu2 lys2-801 trp1Δ63 Ade2+* | S288C | this study |
