# Supplementary figures and images for: The motor domain of the kinesin Kip2 promotes microtubule polymerization at microtubule tips
Source: J Cell Biol. 2023 Apr 24;222(7):e202110126. doi: 10.1083/jcb.202110126 (PMC10130750; doi:10.1083/jcb.202110126)

Kip2 alone   Kip2 + MT   Kip2 + MT   Kip2 alone   MT control  
75 mM   no Salt   75 mM   no Salt  
P   S   P   S   P   S   P   S   P

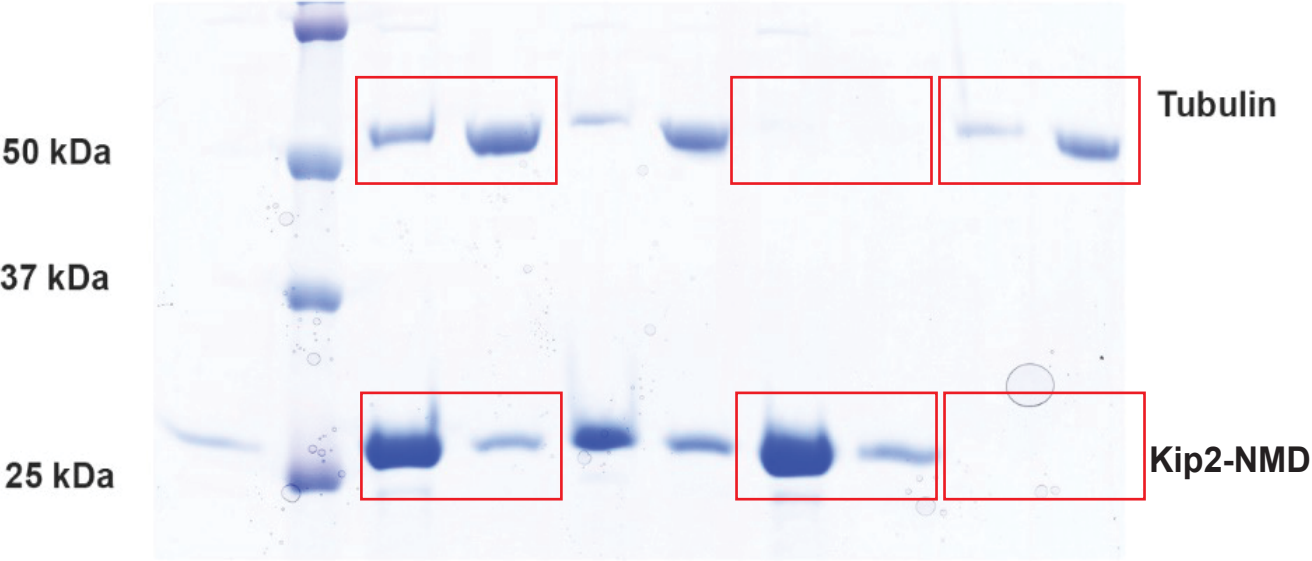

Supplement: SourceData F2 — is the source file for Fig. 2. [file JCB_202110126_SourceDataF2.pdf]

1 Kip2-NMD

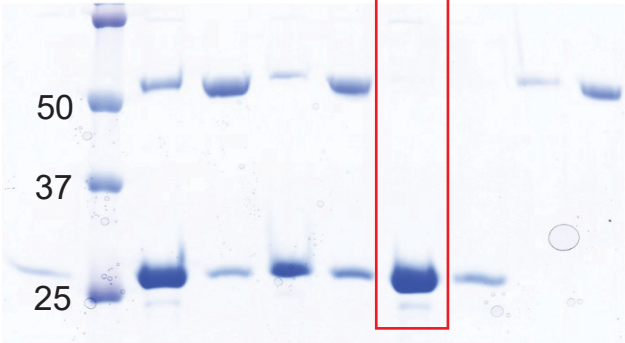

2 BIK1-CC

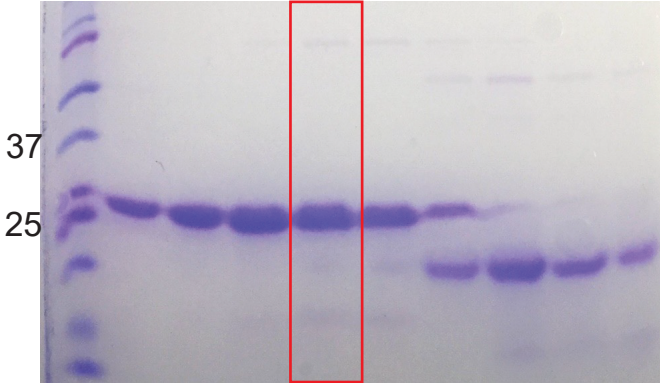

4 MBP-Kip2-MD-mCherry  
3 Tubulin

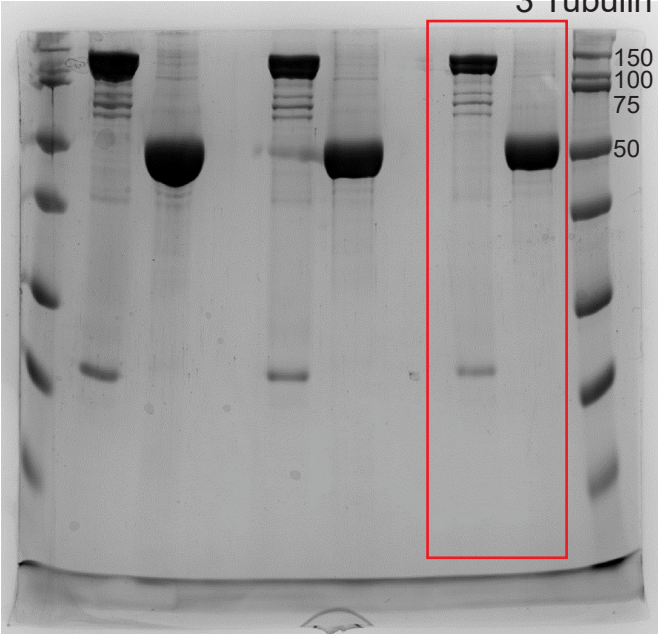

5 MBP-Kip2-MD-P1-mCherry

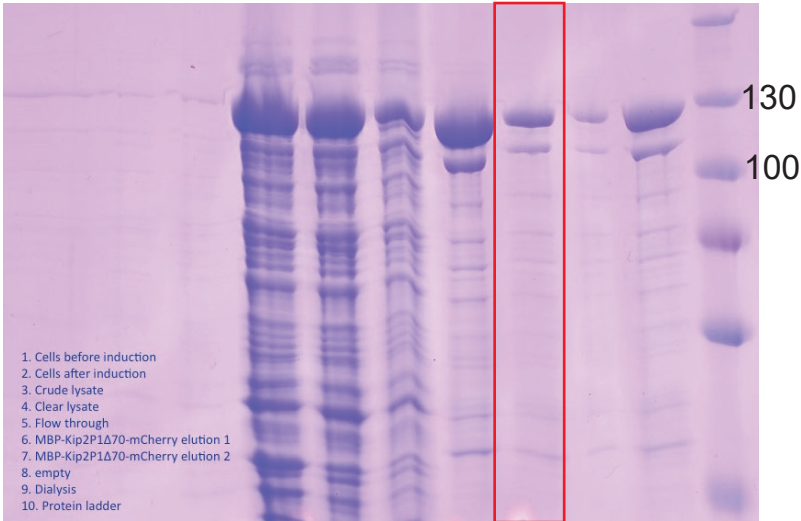

6 Kip2-WT, 7 Kip2-P1-

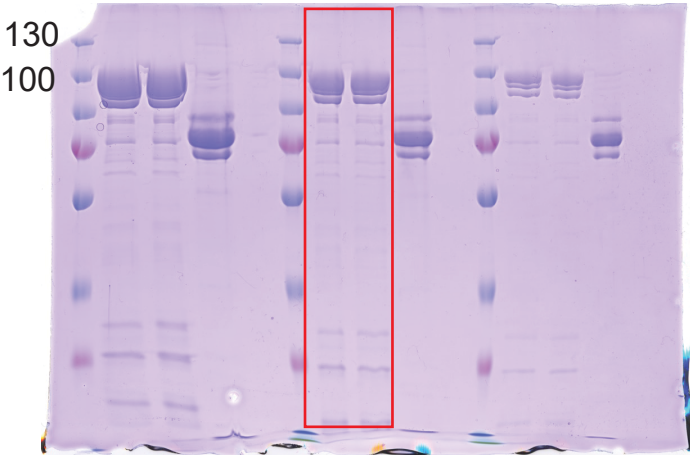

Supplement: SourceData FS1 — is the source file for Fig. S1. [file JCB_202110126_SourceDataFS1.pdf]

kDa  
150 —  
100 —  
75 —

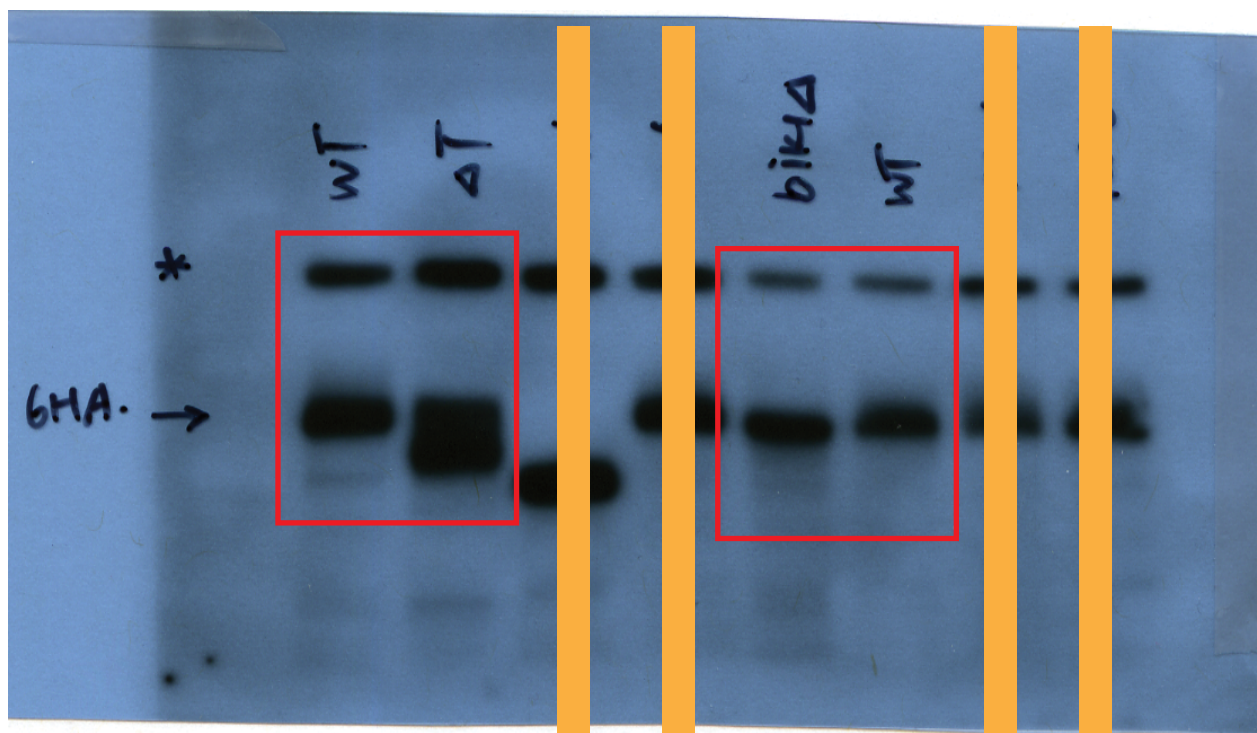

S2D left panel

reversed to be  
S2D right panel

Supplement: SourceData FS2 — is the source file for Fig. S2. [file JCB_202110126_SourceDataFS2.pdf]
